# Additive effects of ceftiofur-neomycin combination against multidrug-resistant pathogenic *Escherichia coli* in a murine infection model

**DOI:** 10.17221/38/2025-VETMED

**Published:** 2026-01-26

**Authors:** Kyung-Hyo Do, Min-Gyu Kim, Da-Hye Ryu, Hyun-Jung Ahn, Su-Bin Kim, You-Kyung Go, So Yeon Kim, Soochong Kim, Seung-Hun Lee, Do-Kyun Kim, Young-Eun Cho, Jihoon Kim, Young Kyung Park, Kounghwa Youn, Hanseul Oh, Kwang-Won Seo

**Affiliations:** ^1^College of Veterinary Medicine, Chungbuk National University, Cheongju, Republic of Korea; ^2^Division of Zoonotic and Vector-Borne Diseases Research, Center for Infectious Diseases Research, Korea National Institute of Health, Cheongju, Republic of Korea; ^3^Korea Zoonosis Research Institute, Jeonbuk National University, Iksan, Republic of Korea; ^4^Department of Food and Nutrition, Kyungkook National University, Andong, Republic of Korea; ^5^School of Integrative Engineering, Chung-Ang University, Seoul, Republic of Korea; ^6^Department of Biomedical Comparative Science, College of Veterinary Medicine, Mississippi State University, Mississippi State, MS, USA; ^7^KHU-KIST Department of Converging Science and Technology, Kyung Hee University, Dongdaemun-gu, Seoul, Republic of Korea

**Keywords:** aminoglycosides, antimicrobial resistance, beta-lactams, combination therapy, whole-genome sequencing

## Abstract

This study aimed to evaluate the therapeutic efficacy of a ceftiofur-neomycin combination against a pathogenic multidrug-resistant *Escherichia coli* strain, KECS-0513, isolated from pigs, using a combination of genomic, *in vitro*, and *in vivo* approaches. The minimum inhibitory concentration, minimum bactericidal concentration, and checkerboard assays were performed. Time–kill assays were used to assess bactericidal activity over time, and a murine intraperitoneal infection model was used to evaluate survival outcomes following treatment with monotherapies or combination regimens. Whole genome sequencing indicated that KECS-0513 harboured multiple resistance genes, including *bla*_TEM-1B_, *sul3*, *aadA12*, *aad(3)-IVa*, *aph(3’)-Ia*, and *aph(4)-Ia*. The resistance genes were localised within a mobile, element-rich plasmid. *In vitro* checkerboard assays revealed an additive interaction between ceftiofur and neomycin (fractional inhibitory concentration index = 1.0), and time–kill experiments demonstrated enhanced and sustained bacterial clearance with combination therapy. *In vivo* infection studies in mice demonstrated that the dual treatment resulted in a substantially higher survival rate (66.7%) compared to treatment with either agent alone (33.3% for each). These results support the practical application of ceftiofur-neomycin combination therapy for controlling swine-associated multidrug-resistant *E. coli* while minimising the risk of resistance emergence.

Pathogenic *Escherichia coli* derived from pigs is a common cause of neonatal diarrhoea, post-weaning enteric disease, and other serious infections that result in substantial economic losses in swine production (Barros et al. 2023). The increasing prevalence of multidrug-resistant *E. coli* strains has significantly impacted the efficacy of conventional treatments. This has led to a necessity for the development of alternative or optimised therapeutic strategies (Do et al. 2023).

Third-generation cephalosporins, such as ceftiofur, are frequently used in veterinary medicine because of their broad-spectrum activity against gram-negative bacteria (Seo et al. 2023). The aminoglycoside antimicrobial, neomycin, is widely used for its potent bactericidal effects in treating gastrointestinal infections in livestock (Do et al. 2023). Although each of these antimicrobials has demonstrated individual efficacy, their combination is frequently used empirically in Korean veterinary practice without systematic evaluation (Do et al. 2023). In contrast to the examination of synergistic combinations involving newly introduced or marginally effective antimicrobials, the objective of this study was to investigate whether an additive interaction between two already-effective, field-approved agents could meaningfully improve therapeutic outcomes. This approach focuses on real-world applicability and provides evidence for the optimisation of existing treatment options in veterinary medicine. Ceftiofur inhibits bacterial cell wall synthesis, while neomycin targets the 30S ribosomal subunit to inhibit protein synthesis (Hayer et al. 2020; Kim et al. 2020). This mechanistic divergence provides a theoretical basis for additive or even synergistic effects (Terbtothakun et al. 2021), which may enhance bacterial clearance and minimise resistance selection pressure. Importantly, both of these antimicrobials have already been extensively used in field settings. However, their combined effect is frequently assumed rather than validated, underscoring the necessity to determine whether additive effects can translate into enhanced outcomes. This approach is consistent with real-world therapeutic practices and contributes to the optimisation of existing antimicrobial treatments based on evidence (Do et al. 2023).

Combination antimicrobial therapy can theoretically enhance bacterial clearance by exploiting distinct mechanisms of action, potentially reducing the required dose of each agent and thereby minimising adverse effects and the risk of further resistance development (Al-Tamimi et al. 2019; Kim et al. 2020). However, few studies have systematically examined whether a ceftiofur-neomycin regimen confers similar advantages in the management of *E.* *coli* infections in swine. Clarifying this question is critical given the ongoing need for novel strategies to safeguard animal health and ensure the sustainable use of antimicrobials. Nevertheless, the use of third-generation cephalosporins, such as ceftiofur, is increasingly restricted in veterinary medicine due to their classification as critically important for human health (Seo et al. 2023). This emphasises the importance of carefully evaluating such combinations within stewardship frameworks.

In this study, the antimicrobial efficacy of a combination of ceftiofur and neomycin was evaluated using a genomically characterised multidrug-resistant *E. coli* strain isolated from swine. The objective of this study was to assess the efficacy of this combination in comparison with monotherapy, in terms of bacterial suppression, prevention of regrowth, and enhancement of host survival. This aims to provide a rational foundation for the practical application of this combination in veterinary medicine.

## MATERIAL AND METHODS

### Bacterial strains

Between 2024 and 2025, 462 *E.* *coli* strains were isolated from pigs exhibiting severe diarrhoea or sudden death with neurological symptoms, as previously described (Do et al. 2023). All isolates were stored in 50% glycerol stock at –70 °C until further characterisation. Of these isolates, a strain designated KECS-0513 was selected for this study because of its high pathogenicity, characterised by the presence of eae, LT, STa, STb, and EAST-1.

### Whole genome sequencing

Genomic DNA was extracted from the KECS-0513 using the Maxwell RSC Faecal Microbiome DNA Kit AS1700 (Promega, Madison, WI, USA) according to the manufacturer’s protocol. Long- and short-read sequencing were conducted in-house. Long reads were obtained using a MinION sequencer (Oxford Nanopore Technologies, Oxford, UK), and short reads were generated using the MiSeq platform (Illumina, San Diego, CA, USA). Raw reads were quality filtered and trimmed using Trimmomatic (v0.39), Filtlong (v0.2.0), and Porechop (v0.2.4). Hybrid *de novo* was performed using Unicycler (v0.4.9b), and the assembly was polished with Pilon (v1.21).

Genome annotation was performed using Bakta (v1.11.0) with the default settings. Multilocus sequence type (MLST) was identified using MLSTFinder. The core genome sequence type (cgST) was determined using cgMLSTFinder. Antimicrobial resistance genes and virulence factors were identified using ResFinder and VirulenceFinder, respectively. Plasmid replicons were detected using PlasmidFinder.

### Minimum inhibitory concentration (MIC) and minimum bactericidal concentration (MBC)

MIC and MBC values of the KECS-0513 were determined using a standard broth microdilution method according to CLSI guidelines (CLSI 2023). Briefly, bacterial suspensions were adjusted to 0.5 McFarland standard in Mueller–Hinton broth (MHB; BD Difco^TM^, Sparks, MD, USA). Two-fold serial dilutions of ceftiofur and neomycin were prepared in 96-well microtitre plates, and 100 μl of the bacterial inoculum was added to each well. After incubation at 37 °C for 20 h, the MIC was recorded as the lowest concentration of the antimicrobial agent that visibly inhibited bacterial growth. For MBC determination, 10 μl from each well showing no visible growth was spot-plated onto LB agar (BD Difco^TM^, Sparks, MD, USA) and incubated at 37 °C for 24 hours. The MBC was defined as the lowest antimicrobial concentration that resulted in no colony growth on the agar plate.

### Checkerboard synergy assay

A checkerboard assay was performed to determine whether ceftiofur and neomycin exerted a synergistic effect against KECS-0513. Two-dimensional, serial two-fold dilutions of both agents were prepared in MHB in 96-well microtitre plates.

After adjusting the bacterial culture to 5 × 10^5^ CFU/ml and inoculating each well, the plates were incubated at 37 °C for 20 hours. Growth inhibition was evaluated visually. The fractional inhibitory concentration index (FICI) was determined by calculating, for each antimicrobial, the ratio of its MIC in combination to its MIC alone, and then adding these ratios for both antimicrobials. A FICI value of 0.5 or lower indicated synergy, values between 0.5 and 4.0 were interpreted as no interaction (additivity or indifference), and values exceeding 4.0 were considered antagonistic (Kim et al. 2020).

### Time–kill assay

Time–kill assays were conducted to evaluate the bactericidal effects of ceftiofur, neomycin, and their combination. Briefly, bacterial cultures (1 × 10^6^ CFU/ml) were exposed to each antimicrobial agent (or both) in MHB at a concentration equivalent to 1 × MIC. Aliquots (100 μl) were removed at 0, 1, 3, 6, and 24 h, serially diluted in phosphate-buffered saline (PBS), and plated on LB agar. After 24 h of incubation at 37 °C, colonies were counted to determine the number of surviving CFU.

### *In* *vivo* infection model

Animal experiments were performed using female BALB/c mice (6-w old), obtained from KoaTech (Gyeonggi-do, Republic of Korea). Mice were acclimatised for one week before experimentation and provided with food and water *ad* *libitum*. All experimental procedures involving animals were conducted with the approval of the Institutional Animal Care and Use Committee (IACUC) of Chungbuk National University (approval no. CBNU-2024-81; approved on 2 September, 2024), and in accordance with the ARRIVE guidelines and Korean animal welfare regulations.

The animals were divided into five groups (*n* = 6 per group): (1) negative control, (2) positive control, (3) neomycin, (4) ceftiofur, and (5) combination treatment. Mice in the negative control group received no challenge and were administered 200 μl of PBS intraperitoneally. Mice in the other four groups were intraperitoneally challenged with 200 μl of KECS-0513 at 1 × 10^9^ CFU/ml.

Thirty minutes after the challenge, mice in the control group received 200 μl of PBS subcutaneously. In contrast, the neomycin group received 200 μl of 512 μg/ml neomycin, the ceftiofur group received 200 μl of 512 μg/ml ceftiofur, and the combination group received 100 μl each of 512 μg/ml neomycin and 512 μg/ml ceftiofur via subcutaneous injection. Survival was monitored for 4 d following infection.

### Statistical analysis

Differences among experimental groups were evaluated using one-way analysis of variance (ANOVA) followed by Duncan’s post-hoc test. The survival data were analysed using the Kaplan–Meier method, and differences among groups were assessed using the log-rank (Mantel–Cox) test. All statistical analyses were performed using SPSS Statistics (v21.0; IBM Corp., Armonk, NY, USA), and a *P*-value of less than 0.05 was considered statistically significant.

## RESULTS

### Whole genome characteristics and plasmid-borne genetic features of KECS-0513

The hybrid whole-genome assembly of KECS-0513 generated five contigs with a total genome length of 5 076 243 bp. Functional annotation identified 4 724 coding sequences (CDSs), 22 rRNA genes, 85 tRNA genes, one tmRNA, and 186 ncRNAs. Additionally, eight pseudogenes and 69 hypothetical proteins were predicted (Table 1). Three plasmid replicons were detected: IncFIB (AP001918) and IncFII (pCoo) were located on contig_2, and another IncFII replicon was identified on contig_3.

**Table 1:** Genomic assembly statistics and functional annotation of *Escherichia coli* KECS-0513

Genomic feature	Value
MLST	2 521
cgST	125 206
	
Total genome size (bp)	5 076 243
Number of contigs	5
	
Annotations	
CDSs	4 724
rRNAs	22
tRNAs	85
tmRNAs	1
ncRNAs	186
Pseudogenes	8
Hypothetical proteins	69
	
Plasmid replicons	IncFIB (AP001918), IncFII (pCoo) on contig 2; IncFII on contig 3
Virulence genotype	fdeC:fimH:lpfA:yehA:yehB:yehC:yehD: astA:eltIAB-4:estap-STa1:estb-STb1: hlyE:tratT:nlpI:traJ:gad:terC:csgA
Antimicrobial resistance genotype	*aph(3’)-Ia:aadA12:aac(3)-IV: bla*_TEM-1B_ *:sul3:aph(4)-Ia*
Antimicrobial-resistant phenotype	ampicillin, gentamicin, kanamycin, streptomycin

bp = base pair; CDS = coding sequences; cgST = core genome sequence type; MLST = multilocus sequence type; ncRNA = non-coding RNAs; rRNA = ribosomal RNA; ST = sequence type; tmRNA = transfer-messenger RNA; tRNA = transfer RNA

The genome contained multiple virulence-associated genes, including adhesins (*fdeC*, *fimH*, *lpfA*), fimbrial cluster genes (*psiA*, *psiB*, *yehA*, *yehD*), and several enterotoxin-related genes (*astA*, *eltlAB*-4, *estap*-*STa1*, *estb-STb1*). Resistance genes conferring antimicrobial resistance to β-lactams (*bla*_TEM-1B_), aminoglycosides (*aph(3’)-Ia*, *aadA12*, *aac(3)-IVa*, *aph(4)-Ia*), and sulphonamides *(sul3*) were also identified. Phenotypic antimicrobial susceptibility tests indicated resistance to ampicillin, gentamicin, kanamycin, and streptomycin.

**Figure 1:**
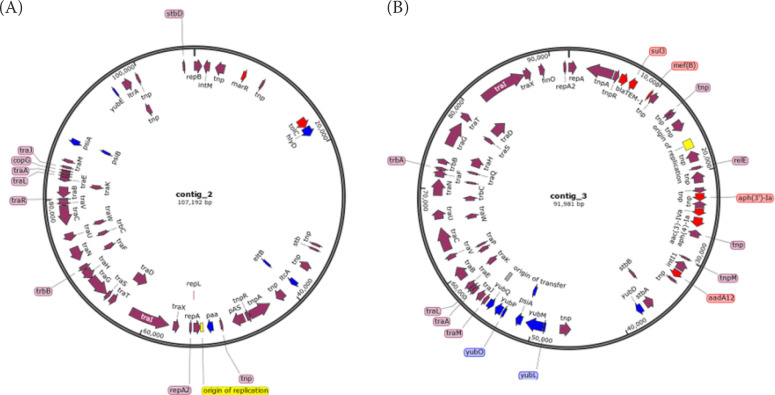
Circular plasmid maps of contig_2 (A) and contig_3 (B) of *Escherichia coli* KECS 0513 Antimicrobial resistance genes are marked in red, virulence-associated genes in blue, and mobile genetic elements in purple. Only functionally annotated coding sequences are shown bp = base pair

Circular maps of contig_2 (107 192 bp) and contig_3 (91 981 bp) indicated distinct functional features associated with each plasmid (Figure 1). Contig_2 encoded a set of conjugation-related genes, including *tra* and *trb* operons, as well as plasmid replication and stabilisation elements, such as *repA* and *copG*. In addition, virulence-associated genes, including *eltB*, *ltcA*, *psiA*, *psiB*, and fimbrial cluster proteins (*yubE* and *yubL*), were also identified.

Contig_3 harboured a dense cluster of antimicrobial resistance genes including *bla*_TEM-1_, *sul3*, *aadA12*, *aac(3)-IVa*, *aph(3’)-Ia*, and *aph(4)-Ia*. These resistance loci were frequently flanked by mobile genetic elements, including transposases (tnp, tnpA, tnpB), the integron integrase (intl1), and the toxin-antitoxin component (relE). Virulence-associated genes, including *psiA*, *psiB*, *yubO*, and *yubL*, were also present.

### MIC, MBC, and checkerboard assay

MIC and MBC values for both neomycin and ceftiofur were determined to be 2 μg/ml each. When tested in combination using checkerboard assays, the MIC of each antimicrobial decreased to 1 μg/ml, yielding an FIC index of 1.0, which indicates an additive effect. No synergistic activity (FIC ≤ 0.5) or antagonism (FIC > 2.0) was observed under the test conditions.

### Time–kill assay

**Figure 2:**
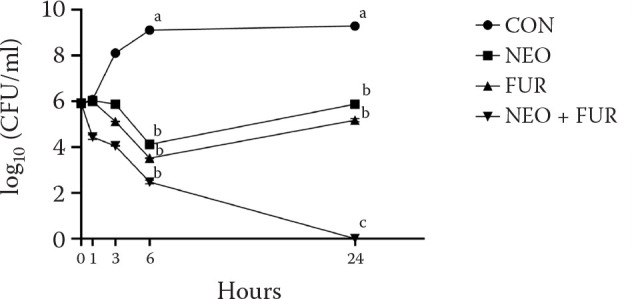
Time–kill curves of *Escherichia coli* KECS-0513 in the presence of neomycin, ceftiofur, or their combination compared to an untreated control Data represent mean ± SD of three independent experiments ^a–c^Different lowercase letters at the same time point indicate statistically significant differences among groups (*P* < 0.05) CFU = colony forming unit; CON = control; FUR = ceftiofur; NEO = neomycin; NEO + FUR = combination of neomycin and ceftiofur

The time–kill curves of KECS-0513 in the presence of neomycin, ceftiofur, or their combination showed significant differences in bacterial growth over 24 hours (Figure 2). In the control culture, bacterial counts increased from 5.92 ± 0.01 log_10_ CFU/ml at zero h to 9.29 ± 0.01 log_10_ CFU/ml at 24 hours. Treatment with neomycin or ceftiofur alone produced an initial reduction in bacterial load – reaching 4.12 ± 0.01 and 3.52 ± 0.04 log_10_ CFU/ml at six h, respectively – followed by partial regrowth by 24 h (5.88 ± 0.01 and 5.18 ± 0.01 log_10_ CFU/ml, respectively). In contrast, the combination of neomycin and ceftiofur led to a more pronounced and sustained decline in bacterial density, falling from 5.92 ± 0.01 log_10_ CFU/ml at zero h to 2.48 ± 0.01 log10 CFU/ml at six h, and reaching undetectable levels (0.00 ± 0.00 log_10_ CFU/ml) by 24 hours.

### Survival rates

**Figure 3:**
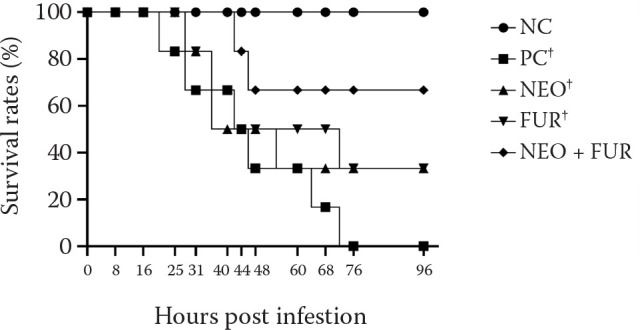
Survival rates of BALB/c mice following intraperitoneal challenge with *Escherichia coli* KECS-0513 A dagger symbol indicates a statistically significant difference compared to the NC FUR = ceftiofur; NC = negative control; NEO = neomycin; NEO + FUR = combination of neomycin and ceftiofur; PC = positive control

Survival curves of BALB/c mice challenged with KECS-0513 and treated under different conditions are shown in Figure 3. The negative control group (NC) maintained 100% survival throughout the 76 h observation period. In contrast, the positive control group (PC) exhibited its first mortality event at 25 h, and survival declined steadily to 0% by 76 h, which was significantly lower than that of the NC (*P *< 0.001). Among the treated groups, mice receiving either neomycin (NEO) or ceftiofur (FUR) alone exhibited moderate protection, with 33.3% survival at 76 hours. Both treatments showed significantly lower survival than the NC (*P* = 0.018 for each) and no significant difference compared with the PC (*P* = 0.361 for NEO, *P* = 0.229 for FUR). The combination therapy group (NEO + FUR) demonstrated a higher overall survival rate, with 66.7% maintained at 76 hours. This rate was not significantly different from the NC (*P* = 0.138), but it was substantially higher than the PC (*P* = 0.023).

## DISCUSSION

This study aimed to evaluate the therapeutic potential of the combination of ceftiofur and neomycin against multidrug-resistant KECS-0513. Whole-genome sequencing of the challenge strain KECS-0513 provided insights into its multidrug resistance and virulence-associated features. The strain carried multiple acquired resistance genes, including *bla*_TEM-1B_, *sul3*, *aadA12*, *aac(3)-IVa*, *aph(3’)-Ia*, and *aph(4)-Ia*, conferring resistance to β-lactams, sulphonamides, and several aminoglycosides. These genotypic findings corresponded well with the observed phenotypic resistance to ampicillin, gentamicin, kanamycin, and streptomycin (Tyson et al. 2015; Plattner et al. 2020; Do et al. 2023; Koster et al. 2023). Regarding ceftiofur, no specific resistance determinants were identified in the genome. For neomycin, although the *aph(3’)-Ia* gene, which encodes an aminoglycoside-modifying enzyme (Plattner et al. 2020), was present, the phenotypic susceptibility testing indicated that the strain remained susceptible to neomycin. This discrepancy highlights that the presence of a resistance gene does not always translate into phenotypic resistance, which may depend on expression levels, regulatory factors, or genetic context.

The resistance genes were clustered within a mobile genetic element-rich region on contig_3, flanked by transposases, an integrase (*intl1*), and toxin-antitoxin stabilisation systems. This structure implies the presence of a composite antimicrobial resistance island with the potential for horizontal gene transfer (Partridge et al. 2018; Rozwandowicz et al. 2018). Contig_2 harboured a complete conjugation system (*tra*, *trb* operons) and virulence-associated loci, including enterotoxin genes (*eltB*, *ltcA*) and fimbrial proteins (*psiA*, *psiB*, *yubE*, *yubL*), suggesting that this plasmid may play a dual role in transmission and pathogenesis (Partridge et al. 2018; Rozwandowicz et al. 2018).

Although ceftiofur- and neomycin-resistance genes were absent, the overall genetic profile of KECS-0513 supported the rationale for exploring combination therapies. In multidrug-resistant backgrounds, using two agents with distinct mechanisms of action may enhance bacterial clearance, minimise treatment failure, and reduce the risk of resistance emergence (Bognar et al. 2024; Morales-Duran et al. 2024). Thus, the genomic characterisation of KECS-0513 not only clarifies its pathogenic and resistance potential but also provides a molecular framework for interpreting the observed therapeutic efficacy of the ceftiofur-neomycin combination.

The present study indicated that both neomycin and ceftiofur exhibited inhibitory and bactericidal activity at a concentration of 2 μg/ml, and that combining them lowered each agent’s MIC to 1 μg/ml. Although combining neomycin and ceftiofur modestly enhanced their activity compared to single-agent use, the effect is not sufficiently potent to be considered synergistic (Al-Tamimi et al. 2019; Kim et al. 2020; Chosidow et al. 2023). The reduction in the MIC for each antimicrobial suggests that dual therapy can achieve bacterial inhibition at lower doses, possibly by reducing toxicity or selection pressures for resistance (Bognar et al. 2024; Morales-Duran et al. 2024). However, the lack of synergy implies that these agents primarily work in parallel, rather than amplifying each other’s mechanisms of action (Bollenbach 2015). From a practical standpoint, an additive combination can benefit animal health programs by reducing the overall required concentration of each agent (Lloyd and Page 2018; Patel et al. 2020), which may be particularly important in livestock settings where controlling antimicrobial use is increasingly emphasised.

The time–kill assay demonstrated that, although neomycin or ceftiofur alone initially reduced the KECS-0513 population, both treatments allowed partial regrowth by 24 hours. In contrast, combining the two antimicrobials led to a profound and lasting decline in bacterial density, ultimately eliminating detectable colonies. These findings suggest that, by targeting bacterial processes through distinct mechanisms, the combination of neomycin and ceftiofur achieves a more sustained bactericidal effect than either agent alone (Hossain et al. 2020; Terbtothakun et al. 2021). Although checkerboard assays indicated an additive interaction, the time–kill data revealed that even additive effects could substantially enhance bacterial clearance over time, particularly under experimental conditions mimicking prolonged drug exposure (Bremmer et al. 2016). The ability to prevent regrowth during the 24 h window implies that combination therapy could reduce the relapse or persistence of infection (Kim et al. 2020; Lazar et al. 2022; Bognar et al. 2024). From a practical perspective, this enhanced and sustained killing activity has potential value in clinical and veterinary settings, where the rapid and thorough eradication of *E. coli* is crucial (Lloyd and Page 2018; Patel et al. 2020). Decreasing the viable counts to undetectable levels may also lower the risk of resistance emergence, as fewer surviving bacteria remain capable of adapting to antimicrobial pressure (Bollenbach 2015; Bremmer et al. 2016; Kim et al. 2020). Moreover, combining agents may allow for optimised dosing protocols, potentially reducing the total amount of each drug required and minimising adverse effects and costs (Lloyd and Page 2018; Kim et al. 2020; Patel et al. 2020).

The *in* *vivo* survival data revealed a stark contrast between the negative control group, which remained fully viable, and the positive control group, which experienced 100% mortality at the end of the observation period. Monotherapy with neomycin or ceftiofur conferred moderate protection (33.3% survival), whereas the combination regimen yielded a substantially higher survival rate (66.7%). These results indicate that both neomycin and ceftiofur can mitigate the lethal effects of KECS-0513 infection in mice; however, their effectiveness was substantially enhanced when the two agents were administered together. Although *in* *vitro* assays have categorised their combined effect as additive, *in* *vivo* data suggest that even an additive interaction can confer a meaningful survival advantage over single-agent treatments. This outcome may be attributed to the broader microbial killing spectrum provided by the combination, as well as the possible reduction in pathogen resistance emergence during active infection (Liu et al. 2020; Bognar et al. 2024). Clinically, this increase in survival emphasises the potential value of using combination therapy against severe *E. coli* infections, especially in veterinary medicine, where rapid and effective control is critical for curbing economic losses and protecting animal welfare (Do et al. 2023). Improved survival also raises the possibility that lower doses of each agent may be sufficient to achieve similar therapeutic outcomes, potentially diminishing the risk of adverse effects or resistance selection associated with higher or more prolonged dosing (Bognar et al. 2024).

This study had certain limitations. Firstly, the use of a single *E. coli* strain limits the generalisability of the findings across diverse pathotypes, sequence types, or resistance genotypes. Secondly, clinical signs beyond mortality were not systematically recorded in the animal experiment, which restricts the depth of clinical interpretation. Thirdly, the clinical applicability of ceftiofur-neomycin combination therapy must be interpreted with caution. Neomycin has been linked to significant toxicity concerns and strict residue limits in meat products, which have led to limitations on its utilisation in food-producing animals across various regions (Lloyd and Page 2018; Patel et al. 2020). Furthermore, ceftiofur is classified as a critically important antimicrobial, and prudent-use guidelines strongly discourage the practice of mass medication in livestock (Seo et al. 2023). Consequently, while the data suggest the therapeutic efficacy of this combination, its practical implementation should be carefully evaluated in the context of antimicrobial stewardship policies and regulatory frameworks.

This study demonstrated that the combination of ceftiofur and neomycin exhibits additive antimicrobial activity against a multidrug-resistant, pathogenic *E. coli* strain. *In vitro* experiments showed reduced MIC values and enhanced bacterial clearance with the combined regimen, whereas *in vivo* administration substantially improved the survival of the challenged mice. These findings support the potential utility of ceftiofur-neomycin combination therapy as a practical strategy to control pathogenic *E. coli* infections in veterinary settings, especially in the context of increasing antimicrobial resistance and the need to preserve therapeutic efficacy through optimised dosing approaches.
